# Impact of IPDE-SQ personality disorders on the healthcare and societal costs of fibromyalgia patients: a cross-sectional study

**DOI:** 10.1186/s12875-016-0464-5

**Published:** 2016-06-01

**Authors:** Laura Gumà-Uriel, M. Teresa Peñarrubia-María, Marta Cerdà-Lafont, Oriol Cunillera-Puertolas, Jesús Almeda-Ortega, Rita Fernández-Vergel, Javier García-Campayo, Juan V. Luciano

**Affiliations:** Mental Health Centre Esplugues, Parc Sanitari Sant Joan de Déu, Rambla Verge de la Mercé, 1 edif. Molí. 08950, Esplugues Sant Boi del Llobregat, Esplugues del Llobregat, Spain; Primary Health Centre Bartomeu Fabrés Anglada, SAP Delta Llobregat, Unitat Docent Costa de Ponent, Institut Català de la Salut, Gavà, Spain; Primary Care Prevention and Health Promotion Research Network, RedIAPP, ISCIII, Madrid, Spain; Teaching, Research, & Innovation Unit, Parc Sanitari Sant Joan de Déu, Sant Boi de Llobregat, Spain; Unitat de Suport a la Recerca, IDIAP Jordi Gol, Primary Health care Department of Costa de Ponent, Institut Català de la Salut, Cornellà de Llobregat, Spain; Department of Psychiatry, Miguel Servet Hospital, Aragon Institute of Health Sciences (I + CS), Zaragoza, Spain; Open University of Catalonia, Barcelona, Spain

**Keywords:** Fibromyalgia syndrome, Personality disorders, Direct costs, Indirect costs

## Abstract

**Background:**

Data is lacking on comorbid personality disorders (PD) and fibromyalgia syndrome (FMS) in terms of prevalence, and associated healthcare and societal costs. The main aim of this study was to assess the prevalence of PD in FMS patients and to analyse whether the presence of comorbid PD is related to worse functional impairment and greater healthcare (medical visits, drug consumption, and medical tests) and societal costs.

**Methods:**

A cross-sectional study was performed using the baseline data of 216 FMS patients participating in a randomized, controlled trial carried out in three primary health care centres situated in the region of Barcelona, Spain. Measurement instruments included the International Personality Disorder Examination - Screening Questionnaire (IPDE-SQ), the Fibromyalgia Impact Questionnaire (FIQ), the Client Service Receipt Inventory (CSRI), and a socio-demographic questionnaire.

**Results:**

Most patients (65 %) had a potential PD according to the IPDE-SQ. The most prevalent PD were the avoidant (41.4 %), obsessive-compulsive (33.1 %), and borderline (27 %). We found statistically significant differences in functional impairment (FIQ scores) between FMS patients with potential PD vs non-PD (59.2 vs 51.1; *p* < 0.001). Multivariate regression analyses revealed that higher FIQ total scores and the presence of potential PD were related to more healthcare costs (primary and specialised care visits).

**Conclusions:**

As expected, PD are frequent comorbid conditions in patients with FMS. Our results suggest that the screening of comorbid PD in patients with FMS might be recommendable in order to detect potential frequent attenders to primary and specialised care.

## Background

Fibromyalgia syndrome (FMS) is a complex entity recognised as an illness by the World Health Organisation (WHO) in 1992 and described in the International Classification of Diseases (ICD-10) under code number M79 [1]. Aetiology is unknown and the course of the illness is chronic. FMS diagnosis is clinical and to date there is no cure, only treatment of symptoms [2]. FMS is characterized by multifocal pain, fatigue, non-restorative sleep, subjective cognitive problems, high levels of distress, and is usually associated with somatic illnesses such as irritable bowel syndrome, migraine, etc. In 1990, the American College of Rheumatology (ACR) established two diagnostic criteria [3]: firstly, the presence of generalised pain for at least three months and, secondly, detection of hypersensitivity in at least 11 of the 18 predefined points on digital application of a force of 4kgs per surface unit. In May 2010, the ACR published new diagnostic criteria [4] that eliminate the tender point examination. FMS diagnosis according to ACR 2010 criteria is based on three key elements: (a) Widespread Pain Index and Symptom Severity Scale above specific cut-off scores, (b) symptoms have been present at a similar level for at least 3 months, and (c) the patient does not have another medical condition that would explain the symptoms.

Prevalence estimates for FMS in the general population have varied from 2 % to 4 % in most studies [5]. Prevalence in primary care consultancies have varied between 5 % and 8 % while rheumatology services report prevalences between 10 % and 20 % [6, 7].

The high prevalence of FMS and its great impact on functioning can lead to considerable direct (health resources) and indirect (temporary or permanent unemployment due to incapacity) economic costs. According to a study by Sicras-Mainar and colleagues [8], the annual cost per patient in Spain is more than 9,000€, approximately 5,000€ more than the reference population. In the U.S., the annual cost of a FMS patient is triple that of the reference population [9].

To be able to offer adequate treatment, it is important to bear in mind the comorbid conditions that patients present with as these can predict quality of life, functional capacity, and the use of health services by these patients [10–12].

Little-studied comorbid disorders in FMS are personality disorders (PD). According to some studies [13–15], between 31 % and 63 % of patients with FMS may have a PD. These studies differ in type of sample and assessment instruments but all agree that the proportion of PD detected in patients with FMS is far greater than that found in the general population; estimated to be between 6 % and 13 % by some studies [16]. Other studies, in contrast, have found a similar percentage of PD to those found in the general population [17]. According to these studies, the most prevalent PD detected in patients with FMS are those categorised as Cluster C disorders in the Diagnostic and Statistical Manual of Mental Disorders (DSM-IV-TR), e.g., avoidant, obsessive-compulsive and, on the other hand, borderline personality disorder found in Cluster B [18]. In a study by Blasco Claros et al. [19], it was concluded that patients with FMS have fewer narcissistic, histrionic, and antisocial personality traits than in the general population.

The aims of this study are, firstly, to assess the prevalence of potential PD in a sample of patients with FMS and, secondly, to determine whether comorbid FMS and PD result in a greater degree of functional deterioration than in patients with FMS only. Finally, to evaluate whether health care costs associated with patients with FMS and PD are higher than those for patients without PD.

## Methods

In the present work, we used the FibroQoL study baseline dataset [20, 21]. The FibroQoL study was a 12-month, randomised controlled trial whose main aim was to assess the effectiveness and cost-utility of a psycho-educational intervention for FMS patients compared with usual care (http://clinicaltrials.gov/show/NCT00550966). A detailed description of the study protocol can be found elsewhere [22].

### Participants

The FibroQoL study had the following inclusion criteria: Patients aged 18–75 years, contactable by telephone, and who met the 1990 ACR criteria for FMS were candidates for inclusion in the study. Patients with cognitive impairment, presence of physical/psychiatric limitations that impeded participation in the study assessments, or life expectancy of less than 12 months were excluded. The sample pool was composed of all patients included in the FMS database at the Viladecans Hospital (Barcelona, Spain), from 2005 to 2008, who were referred by general practitioners from three Primary Health Centres to the rheumatology service to confirm a diagnosis of FMS.

### Procedure

**Fig. 1:**
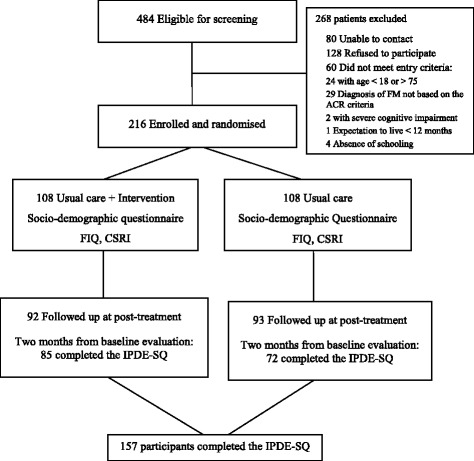
Flow diagram of the participants in the FibroQoL study who completed the IPDE-SQ

The potential participants were screened through an initial phone interview and were provided a general overview of the study. A research assistant then made an appointment for those patients that agreed to participate in the study. Finally, the research assistant, who was not involved with the treatment and was blind to group allocation, performed all the face-to-face interviews. Data were collected at baseline, upon completion of the intervention, (2 month) and at 6 and 12-month follow-up. For the present work, only baseline data were analysed. IPDE-SQ data were collected upon completion of the intervention. Figure 1 illustrates the flow of participants through this study.

### Instruments

#### Socio-demographic questionnaire

Collected information on the following variables: gender, date of birth, marital status, living arrangements, educational level, and work status.

#### Fibromyalgia impact questionnaire

(FIQ) [23, 24]. Measure of functional status that includes 10 items. The first item addresses the ability to perform physical tasks. In the Spanish version, this physical function item contains 9 sub-items rather than the original 10. Items 2 and 3 ask the patient to mark the number of days they felt well and the number of days they were unable to work because of FMS symptoms. Items 4 to 10, inclusive, rate work difficulty, pain, fatigue, morning tiredness, stiffness, anxiety, and depression. The FIQ has a maximum total score of 80 (excluding job-related items), with higher scores indicating greater impact.

#### Client service receipt inventory—adapted

(CSRI) [25, 26]. The CSRI variant used in this study was designed to collect retrospective data on medication and service receipt: *Medication*. A profile of the patient’s use of all prescribed medications was requested. *Service receipt*. The main categories were: emergency service (total visits), general medical inpatient hospital admissions (total days); outpatient health care services (total visits to general practitioner, nurse, social worker, psychologist, and other community health care professionals). Patients were also asked about type and number of medical tests administered. The CSRI was administered in all assessments with varying time frames (the previous 3 months at baseline).

### Descriptions of costing procedure

Direct health care costs were calculated by adding the costs derived from medication consumption, medical tests, and use of health-related services. The cost of medication was calculated by determining the price per milligram during the study, according to the Vademecum International (Red Book; edition 2008), and included value-added tax. Total costs of medications were calculated by multiplying the price per milligram by the daily dose in milligrams and the number of days receiving such treatment. The main source of unit cost data related to medical tests and use of health services was provided by the official tariffs published in the Official Bulletin of the Catalonia Government for 2008. Indirect costs were calculated by multiplying the number of days on sick leave by the minimum daily wage in Spain for 2008. Finally, total costs were calculated by adding direct and indirect costs. The unit costs are expressed in Euros (€) based on 2008 prices.

#### International Personality Disorder Examination – Screening Questionnaire (IPDE-SQ)

PD screening was performed using the DSM-IV version of the IPDE-SQ [27, 28]. The IPDE-SQ is a brief, efficient screening questionnaire. It consists of 77 true/false self-report items intended to assess PD. Its psychometric properties have not been studied in depth, but there are some studies with small samples that have found a sensitivity near 1.00 and a specificity around 0.60 [29, 30]. The IPDE-SQ manual recommends a change in the cut-off depending on the population or study aim. To increase specificity, we used a more conservative cut-off point; increasing the cut-off by two points (from ≥ 3 to ≥ 5). This procedure has been used in previous studies [31–35].

### Statistical analyses

Characteristics of the sample assessed with the IPDE-SQ were compared to those of the FibroQoL study sample that was not assessed with the IPDE-SQ to test the representativeness of the former. Descriptive statistics were presented in terms of means and standard deviations for continuous variables and absolute and relative frequencies for categorical variables. For each type of PD (according to IPDE-SQ) the percentage over the total of PD diagnoses was obtained (in addition to the percentage over the sample). Bivariate analysis of continuous outcomes was assessed by computing means and SDs for each response category of the independent variables and performing a *t*-test or ANOVA to test for possible differences between categories. Finally, multivariate regression models were constructed for global direct costs, medications, tests, primary care services and specialised services, taking as explanatory variables age, years with diagnosis and FIQ score. Given the positively-skewed distributions (truncated at 0) of the dependent variables, Tobit models were adjusted. As our interest lay in the observed variables rather than the latent variables that Tobit models regress, Tobit coefficients were transformed [36] and 95 % CIs bootstrapped.

## Results

### Presence of potential PD

Of the 216 participants in the FibroQol study, 157 (72.7 %) completed the IPDE-SQ and of these, 102 (65.0 %) had a possible PD. The most prevalent PD in the studied sample were avoidant personality disorder (41.4 %), obsessive-compulsive personality disorder (33.1 %) and borderline personality disorder (27 %). Table 1 shows the distribution of PD types and the clusters they belong to, according to the IPDE-SQ results. There was an ample percentage of participants with more than one possible PD diagnosis. Only 19.7 % of patients had one potential PD while 20.4 % of the sample presented three or more (see Table 2). Multiple overlaps were also observed with only 41 (40.2 %) of the 102 patients having potential PD from the same cluster, 49 (48 %) having possible PD from 2 different clusters and 12 (11.8 %) from three different clusters.

**Table 1:** Potential personality disorders (PD) derived from the IPDE-SQ scores

Type of PD	Cluster	N	% over PD diagnosis	% over the sample
Avoidant	C	65	27.3	41.4
Obsessive-compulsive	C	52	21.8	33.1
Borderline	B	43	18.1	27.4
Schizoid	A	24	10.1	15.3
Schizotypal	A	14	5.9	8.9
Dependent	C	13	5.5	8.3
Histrionic	B	13	5.5	8.3
Narcissistic	B	8	3.4	5.1
Paranoid	A	6	2.5	3.8
Antisocial	A	0	0.0	0.0

**Table 2:** Number of personality disorders (PD) for FMS patients and for FMS patients with at least 1 potential PD diagnosis, derived from the IPDE-SQ scores

	Patients		Diagnosis	
Number PD	N	%	N	%
0	55	35.0	0	0
1	31	19.7	31	13.0
2	32	20.4	64	26.9
3	23	14.6	69	30
4	9	5.7	36	15.1
5	5	3.2	25	10.5
6	1	0.6	6	2.5
7	1	0.6	7	2.9
Total	157	100.00	238	100.00

With respect to the interaction between the potential PD present in a single patient from distinct clusters, only 3 patients of the 35 with a PD diagnosis of Cluster A had no diagnoses from other clusters. Eight of 51 patients had a Cluster B diagnosis only, and 30 of the 89 had a Cluster C diagnosis only. The least frequent interactions were Cluster A with Cluster B (14 of 102; 13.7 %) and the most frequent were Cluster B with Cluster C (41 of 102; 40.2 %) (Online Resource 1).

No statistically significant differences were found in either socio-demographic or health cost variables between those patients who were assessed with the IPDE-SQ and those who were not. We only found marginally significant differences in functional status (FIQ total score) between the patients that were evaluated with the IPDE-SQ and those that were not evaluated, with the latter being slightly more deteriorated (Online Resource 2).

### Relationship between potential PD and functional status (FIQ score)

Table 3 shows, from the bivariate analysis, how the functional status of people with FMS, measured using the FIQ, did not vary with respect to age, gender, years with FMS diagnosis, work status, marital status or living arrangements. There were differences according to educational level as those with more than primary education were slightly less deteriorated than those with primary education only.

**Table 3:** Bivariate analysis of FIQ scores according to socio-demographic characteristics and potential personality disorders (PD)

	N	FIQ	*p*
Age			0.75
[33, 45]	24	53.5 (15.6)	
(45,50]	18	54.2 (10.5)	
(50,55]	34	57.8 (14.0)	
(55,60]	35	56.2 (13.9)	
(60,65]	31	57.3 (11.0)	
(65,75]	15	58.5 (10.5)	
Gender			0.55
Woman	154	56.5 (13.0)	
Men	3	50.8 (13.6)	
Years with diagnosis			0.48
[0,5]	24	52.1 (16.7)	
(5,10]	32	56.2 (12.2)	
(10,20]	34	56.9 (11.6)	
(20,50]	25	57.4 (13.9)	
Working status			0.27
Employed	54	55.0 (13.4)	
Non employed	91	57.4 (12.5)	
Marital status			0.14
With partner	126	55.7 (13.3)	
Without partner	31	59.1 (11.1)	
Living arrangement			0.57
Live alone	146	56.5 (13.0)	
Live with others	11	54.3 (12.1)	
Educational level			**0.04**
> Primary school	46	53.0 (12.5)	
≤ Primary school	111	57.7 (12.9)	
PD			**<0.01**
No	55	51.1 (13.2)	
Yes	102	59.2 (12.0)	

Significant differences were found on the FIQ depending on whether the patient had a potential PD or not (*p* < 0.001). The FIQ value went from 51.1 (FMS without PD) to 59.2 (FMS + PD). The difference between the two groups on the FIQ was 8.1 points, which is considered a minimal clinically important difference^1^ [37]. The results of the multivariate regression model for the FIQ total score showed that presence of a possible PD is a variable that significantly predicts FIQ score (potential PD *B* = 7.55, *p* = 0.003), while variables such as age or years with diagnosis were not significant (*p* > 0.05).

### Relationship between potential PD and direct/indirect costs (CSRI)

According to the bivariate analysis, direct costs varied in line with the FIQ and depending on whether a potential PD was present or not. The higher the FIQ score, i.e., the greater the functional deterioration, the higher the associated direct costs (*p* < 0.001). The presence of a possible PD was also related to higher direct costs (*p* < 0.008). However, these variables were not significantly associated with indirect costs (Online Resource 3).

Table 4 shows a breakdown of direct costs (medical tests, medication, primary care assistance and specialised care). It can be seen that the FIQ score does have a significant effect on primary health care service and specialised care costs. The relationship between the FIQ score and costs associated with medical tests is also close to significance. Having a potential PD also has an effect on the same factors, increasing primary care and specialised care costs.

**Table 4:** Bivariate comparison between specific direct costs (€), FIQ scores, and potential personality disorders (PD)

	N	Medication (M, SD)	Tests (M, SD)	Primary care services (M, SD)	Specialist services (M, SD)
**FIQ**		*p* = 0.24	*p* = 0.06	***p*** = 0.02*	***p*** < 0.01*
[15,40]	20	84.5 (113.1)	84.9 (106.1)	81.0 (82.8)	90.4 (83.0)
(40,50]	25	100.1 (133.8)	125.7 (137.3)	123.5 (154.3)	145.2 (196.1)
(50,60]	41	229.9 (544.7)	131.4 (134.8)	103.6 (76.4)	148.5 (109.9)
(60,70]	47	222.3 (284.0)	186.9 (160.6)	183.9 (218.1)	399.6 (532.9)
(70,90]	24	110.4 (208.2)	271.7 (493.5)	240.1 (316.9)	332.5 (358.2)
**PD** ^b^		*p* = 0.24	*p* = 0.71	***p*** = 0.03*	***P*** < 0.01*
No	55	132.3 (236.1)	154.3 (155.7)	110.6 (114.5)	156.7 (148.0)
Yes	102	190.6 (382.1)	167.1 (270.2)	169.4 (223.6)	290.9 (423.8)

Note: For each cost variable the table presents its mean (M) and standard deviation (SD)

*p-value for the *t*-test/ANOVA for differences in costs

^b^Comparisons FMS + PD vs. FMS without PD. We computed Cohen’s *d* (rule of thumb: 0.20 = small; 0.50 = medium; 0.80 = large). The magnitude of the effect size was small to medium in both primary care services (*d* = 0.31) and in specialist services (*d* = 0.38)

In contrast, the socio-demographic variables do not have an impact on the distinct direct costs assessed (Online Resource 4).

Tables 5 and 6 show the Tobit multivariate regression models that were performed to determine which factors were associated with specific direct costs. The FIQ score has a significant relationship with direct costs in patients with and without a possible PD. In other words, the higher the FIQ score, the higher the costs. If we consider the intercept, which indicates the mean cost of the mean patient, we observe that costs associated with patients with potential PD are higher, with a confidence interval with values far higher than those for patients without potential PD.

**Table 5:** Multivariate Tobit regression models on Direct Costs (Medication, Tests)

	Direct costs		Medication		Tests	
	No PD	PD	No PD	PD	No PD	PD
(Intercept)	**583.4** (478.0, 692.9)	**785.2** (643.2, 949.8)	98.9 (50.3, 186.1)	155.2 (100.7, 241.7)	125.2 (60.6, 204.1)	136.1 (90.1, 179.5)
Age	−3.1 (−14.8, 7.2)	**−25.2** ^a^ (−40.9, −12.4)	−4.0 (−11.6, 1.5)	−5.1 (−12.9, 0.7)	0.6 (−6.2, 6.9)	−5.5 (−11.1, −0.8)
Years with diagnosis	2.4 (−6.9, 12.6)	4.1 (−15.5, 21.6)	3.2 (−1.6, 7.6)	4.6 (−3.3, 17.0)	−1.3 (−6.9, 4.8)	−0.8 (−8.3, 4.2)
FIQ	**12.3** ^a^ (6.3, 20.2)	**20.8** ^a^ (7.1, 39.0)	2.7 (−0.8, 9.0)	1.6 (−3.2, 5.6)	**3.3** ^a^ (0.2, 6.6)	**5.2** ^a^ (0.9, 12.7)

Note: Transformed Tobit coefficients with bootstrapped 95 % CIs (^a^indicating statistically significant coefficients –alpha = 0.05-). Explanatory variables centred to the sample mean. Accordingly, Intercept approximates the cost of a “mean individual” (aged 55.22, with 14.2 years with diagnosis, and a FIQ score of 56.36). For clarity, significant results are in bold

**Table 6:** Multivariate Tobit regression models on Direct Costs (Primary and Specialist Services)

	Primary care services		Specialist services	
	No PD	PD	No PD	PD
(Intercept)--	**87.2** (66.5, 113.2)	**138.3** (106.5, 186.4)	**146.2** (107.5, 184.7)	**214.3** (151.5, 298.7)
Age	−1.2 (−3.4, 0.9)	−3.0 (−8.2, 0.7)	**3.9** ^a^ (0.5, 7.0)	**−9.3** ^a^ (−16.6, −2.4)
Years with diagnosis	2.8 (−0.9, 8.0)	−1.2 (−6.0, 3.6)	−2.6 (−6.2, 0.6)	0.8 (−8.4, 7.9)
FIQ	1.7 (−0.2, 3.0)	**4.8** ^a^ (1.2, 10.0)	**3.9** ^a^ (1.8, 6.2)	**9.0** ^a^ (3.4, 16.3)

Note: Transformed Tobit coefficients with bootstrapped 95 % CIs (^a^indicating statistically significant coefficients –alpha = 0.05-). Explanatory variables centred to the sample mean. Accordingly, Intercept approximates the cost of a “mean individual” (aged 55.22, with 14.2 years with diagnosis, and a FIQ score of 56.36). For clarity, significant results are in bold

Regarding medical tests, the FIQ score is significant for patients with and without possible PD. For costs related to primary care, the FIQ is only significant in the case of potential PD. If no PD is present, a higher FIQ score is not associated with higher costs. A great difference can be observed between the two groups with respect to the intercept where FMS patients with a potential PD show more costs related to primary care visits. In specialised care great differences are again seen between patients with possible PD and those without. In both cases, the FIQ score has an impact on costs. Differences were also found between the two groups with respect to age: in the group of patients without PD, the older the individual, the higher the costs, while in the potential PD group, the older the patient, the lower the costs. With regard to the ‘medical tests’ variable, no significant association was found.

## Discussion

This study focused on the presence of potential PD in patients with FMS and found that 65 % of patients met criteria for a potential PD according to the IPDE-SQ. This prevalence may seem high but does not differ much from data found in the literature. In a study carried out in Brazil [13], 47 women with FMS were assessed through a clinical interview in a hospital setting. Some 63 % were diagnosed with a PD (vs. 8 % of controls). Another study [14] performed in France evaluated a sample of 30 outpatients with FMS using the SCID-II instrument. A total of 46.7 % were diagnosed with at least one PD with the most prevalent disorders being obsessive-compulsive (30 %), borderline (16.7 %), and depressive (16.7 %). More recently, in a study conducted in Turkey, the SCID-II was administered to 103 patients with FMS in a hospital rheumatology unit (vs. 83 controls). The percentage of participants with PD was lower; 31.1 % (vs. 13.3 % in controls). Regarding the type of PD, 23 % were obsessive-compulsive, 10 % avoidant and 11 % passive-aggressive [15].

Our study used the IPDE-SQ for assessment of PD. The developers of the instrument describe it as a screening tool with high sensitivity and low specificity. Various studies have changed the cut-off point to increase specificity. For instance, Lewin and colleagues explored whether it was better to vary the cut-off point according to the number of diagnostic criteria corresponding to each PD in the ICD-10, i.e., a cut-off point of 3, 4 or 5 depending on the type of PD. This formula appears to give good results [32]. Fernández del Río attempted to replicate these results, comparing the IPDE-SQ with the IPDE using two distinct statistical methods, but did not find conclusive results as discrepancies arose depending on the PD and the method selected. The cut-off points were 4/5, except in the case of antisocial personality disorder, which only required a cut-off point of 3 [33].

In our sample, the presence of potential PD taking a reference cut-off point of 3 was extremely high (97.5 %). This is consistent with the results of another study carried out in primary care which also used the IPDE-SQ with the same cut-off point (96.7 %) [38]. As such, based on previous studies, the decision to use a cut-off point of 5 in our study aimed to offer a more conservative perspective. With this higher cut-off point, our results show that 65 % of patients with FMS suffer from a possible PD with the most prevalent being the avoidant (41.4 %), obsessive-compulsive (33.1 %) and borderline (27.4 %). Another important aspect is that only 20 % presented a single possible PD while, among the other patients, double or triple possible diagnoses were made. Multiple diagnoses are, in general, common in studies of PD [39].

It is important to stress that all PD apart from the antisocial are represented. The least frequent are the paranoid and the narcissistic. These three types of PD are characterised by a lack of empathy and an aggressive interpersonal style. Various studies have attempted to determine whether FMS sufferers have any distinctive traits with respect to the general population. In the meta-analysis carried out by Malin [40], it was shown that there is no clear evidence that this is the case. It should be borne in mind that the the assessment of PD through questionnaires is somewhat controversial due to the limitations involved [41]. Nevertheless, in almost all the cited studies, the prevalence of PD is higher in the population with FMS than in the general population (an estimated 6–10 % of people in the community have a PD) [16].

In the case of patients with a potential PD, the global FIQ score is significantly higher and, as such, implies greater functional deterioration. This finding is consistent with those observed in other studies on the effects of PD on functional status and quality of life. These studies gather data on difficulties in maintaining instrumental roles, productive roles, or good social functioning [16, 42]. Chen [43], in a longitudinal study, showed how quality of life is more deteriorated in adults who have suffered from a PD from a young age (Axis II of DSM-IV) than in those who present Axis I mental disorders.

Focusing on direct costs, the analysis indicated that patients with a potential PD report more costs related to primary and specialised care visits than FMS patients without a potential PD. No differences were found regarding costs associated with consumption of medication or complementary tests, nor were differences detected with respect to indirect costs.

Differences were observed between the group of patients with and without potential PD with regard to age. In the group without potential PD, the older the patient, the higher the costs while, in contrast, in the potential PD group, the older the patient, the lower the costs. This may be because patients with potential PD, being more severe, are referred more promptly to specialised services while the opposite could be the case for those without PD. These data are in accord with those found in some studies which analysed the relationship between the number and type of medical comorbidities in FMS, and functional disability and use of medical services. It has been observed that the number of comorbidities is one of the factors which explains medical costs on FMS [12, 44]. Psychiatric comorbidities determine medical service use to a greater extent than so-called functional illnesses [10]. In a study of the factors which have an impact on FMS costs, it was shown that comorbid illnesses contribute to direct costs in such a way that an increase of 20 % is seen for each additional comorbidity [45].

It should be pointed out that people with a PD (without FMS) tend to use more medical services than those without a PD [46]. According to Olssøn [47], having a PD is associated with more frequent occurence of somatic illnesses and patients report more muscular pain, asthma, FMS, and alcohol problems than controls. Moreover, they tend to take non-prescribed analgesic and anti-depressant medication, consult their GP more, are referred less frequently to somatic specialists, and are less satisfied with their last visit to their GP.

As we pointed out in a previous study using FibroQoL data [48], an important limitation is that the recruited participants are not representative of all Spanish regions and probably of the Spanish general population with FMS. Our patients were voluntarily participating in a RCT, so they were highly motivated. They were the type of patients that present to rheumatology services, agree to participate in research projects, and meet specific inclusion criteria. In the present work, this limitation is even greater, because our sample only reflects the results of 157 out of 484 initially screened patients. Therefore, it is likely that the amount of PD being assessed are much greater than would be seen in a study of FMS in the general population (over-estimation and channeling bias). Replicating our findings in a community primary care setting would be a good follow-up to this investigation.

Another important limitation of the present work is the use of a screening instrument (IPDE-SQ) instead of a structured clinical interview. In other words, there is a risk of heightened false positives. Due to problems concerning the validity of the usual cut-off of three for the IPDE-SQ in some populations (e.g. prisoners, adult individuals seeking speech treatment for stuttering, etc.), a cut-off point of responding affirmatively to five or more answers was applied here. We think that this cut-off modification partially resolves the problem of over-diagnosis because some previous studies have indicated that the presence of Axis II disorders is largely consistent when comparing adjusted screen scores with structured clinical interviews [49].

We consider it important to address in future studies how having a comorbid PD affects the effectiveness of pharmacological and non-pharmacoloical treatments for FMS. We have not found any studies that deal with this aspect although there are studies that have examined how having a PD affects the severity and treatment of Axis I mental disorders. In a 2003 review, it was concluded that PD could cause a poor result in the treatment of an Axis I disorder [50]. Other studies demonstrate the need to vary the type of treatment for major depression according to whether a PD is present or not [51]. It has also been observed that suffering from a PD increases the possibility of abandoning treatment for an Axis I disorder if the PD is not also treated [52].

There is consensus among experts that FMS is far from being a homogeneous clinical entity and that subgroups of patients may be identifiable [53]. Data from the present study suggests that routine screening in primary health care services of comorbid PD among FMS patients might be a cost-effective strategy. This subtype of FMS patients with comorbid PD significantly consumes more healthcare resources, mainly primary care and specialized services. The early detection of a potential PD might set in motion tailored treatments addressed to this subgroup of patients, which are very likely to be treatment resistant. If “one-size fits-all” treatment approaches have high probability of being not effective in the case of FMS due to its heterogeneity, managing FMS patients with comorbid PD in the same way that those without PD is even more likely to be unsuccessful. It is of paramount importance to provide information and training to primary care providers in management strategies for this complex syndrome given that most FMS patients are attended and monitored in this level of care. In fact, the management of FMS in primary health care and the need for training and information for primary care providers is a current topic of interest among experts in the field [54, 55].

## Conclusion

In conclusion, our study indicates that a high percentage of patients with FMS present a potential comorbid PD. Among the most prevalent PD are the avoidant, obsessive-compulsive and borderline disorders. Patients with FMS and concomitant possible PD have worse functional status and higher direct costs, especially in terms of visits to primary healthcare and specialists.

## Data availability

Data available on request.

## Abbreviations

ACR, American College of Rheumatology; CSRI, Client Service Receipt Inventory; DSM-IV-TR, Fourth Edition of the Diagnostic and Statistical Manual of Mental Disorders (Revised); FIQ, Fibromyalgia Impact Questionnaire; FMS, Fibromyalgia syndrome; ICD-10, International Classification of Diseases; IPDE-SQ, International Personality Disorder Examination - Screening Questionnaire; PD, Personality disorders; WHO, World Health Organisation.

## Footnotes

1 In order to test whether the 7 FMS patients with more than 5 PDs might have biased our results, we also computed the FIQ medians and the interquartile ranges (IQR). The median is a statistic that is not influenced by few large values in the data and the IQR is a measure of dispersion that accompanies the median. It is the distance between the upper and lower quartiles. FMS + PDs [FIQ Median = 62.68 (51.94, 67.87)] vs FMS without PDs [FIQ Median = 51.33 (42.95, 60.59)]; the difference is significant at *p* < 0.001.
